# A Transient-Enhanced Voltage Regulator with Stability and Power-Supply-Rejection Boosting

**DOI:** 10.1186/s11671-019-3215-4

**Published:** 2019-12-05

**Authors:** Yue Shi, Anqi Wang, Jianwen Cao, Zekun Zhou

**Affiliations:** 1 College of Communication Engineering, Chengdu University of Information Technology, Chengdu, 610225 China; 2 State key Laboratory of Electronic Thin Films and Integrated Devices, University of Electronic Science and Technology of China, Chengdu, 610054 China

**Keywords:** Voltage regulator, Overcurrent protection, High stability, Self-power, Dynamic load

## Abstract

A high-stability voltage regulator (VR) is proposed in this paper, which integrates transient enhancement and overcurrent protection (OCP). Taken into consideration the performance and area advantages of low-voltage devices, most control parts of proposed VR are supplied by the regulated output voltage, which forms self-power technique (SPT) with power supply rejection (PSR) boosting. Besides, the stability and transient response are enhanced by dynamic load technique (DLT). An embedded overcurrent feedback loop is also adopted to protect the presented VR from damage under overload situations. The proposed VR is implemented in a standard 350 nm BCD technology, whose results indicate the VR can steadily work with 5.5–30 V input voltage, 0–30 mA load range, and 0.1–3.3 μF output capacitor. A 2.98 μV/V line regulation and a 0.233 mV/mA load regulation are achieved with a 40 mA current limiting. The PSR is better than − 64 dB up to 10 MHz with a 0.1 μF output capacitor.

## Introduction

In modern nanometer-scale system on chip (SoC) designs, different sub-blocks usually require different supply rails to achieve some specific functions. Besides, the whole SoC system may need to operate under a wide range of input voltage and still provide high performance unaffected by the supply conditions changing [1]. Thus, wide input voltage range voltage regulator (VR) implemented in nanometer-scale technology can be one of the most suitable candidates for this kind of applications. Compared with its switching counterpart, such as switching regulator and charge pump, linear VR has the advantage of high precision, low output noise, and compact size. Most of linear VRs perform their voltage regulating function with a single voltage supply, but only a few can achieve the combination of wide power supply range, low noise, fast transient, high load capability, and extra protection features [2–4].

To realize a wide power supply range, the utilization of transistors that can stand high voltage pressure is necessary. However, this kind of transistors usually occupies more area and has worse performance in comparison with the standard transistors. Two of the existing solutions to reduce the use of high voltage transistors are the preregulator method [5, 6] and the stacked low voltage transistors method [7, 8]. The former uses an additional preregulator to provide an internal supply voltage for the core regulator. The latter implements well-designed stacked low voltage transistors to maintain the terminal voltages of transistors within technology limit [7]. However, these methods limit the voltage headroom. This paper adopts the self-power technique (SPT) to achieve a wide power supply range, which means most core modules in regulation loop are supplied by the regulated output voltage of proposed VR [9]. Without any additional circuit, the performance of VR can be improved by SPT and also reduce the number of high voltage devices.

By using SPT, the first gain stage of error amplifier (EA) is supplied by the regulated output voltage of proposed VR. By carefully design the second stage of EA, the high frequency noise of the input voltage has little impact on the output voltage of the EA. Furthermore, the N-type power transistor architecture is adopted in this paper. Therefore, the proposed regulator has high power supply rejection (PSR) and good noise performance [10–15].

Fast transient response is also an important index to measure the performance of VRs [16–20], which is usually achieved by adding an extra speedup loop [21]. In this paper, the transient enhancement is realized by dynamic load technique (DLT). During transient response procedure, DLT introduces an additional current changed correspondingly with load conditions to act as a dynamic load, by which the overshoot and undershoot of regulated output voltage can be suppressed [9].

With the help of DLT, the loop stability within a wide load range is strengthened because the additional load current can reduce the variation of the non-dominant pole position. To make the loop stable, miller compensation with nulling resistor is also used for generating a low frequency pole and an extra zero to compensate the output pole. Besides, a resistor series with the output capacitor introduces another zero to compensate the pole generated by the parasitic gate capacitance of the power transistor [6, 9, 22, 23].

Finally, an embedded current limiting loop is designed to avoid overcurrent damage and improve reliability of the proposed VR [9, 24–27].

This paper is structured as follows. The principle and mechanism of proposed VR are illustrated in the “Method” section, including the overcurrent protection (OCP) circuit, the DLT and transient enhancement circuit, the stability boosting method, and the PSR analysis. The performance results and comparisons with other related published literatures are shown in the “Results and Discussion” Section. The “Conclusion” section draws the conclusion of proposed VR.

## Method

**Fig. 1:**
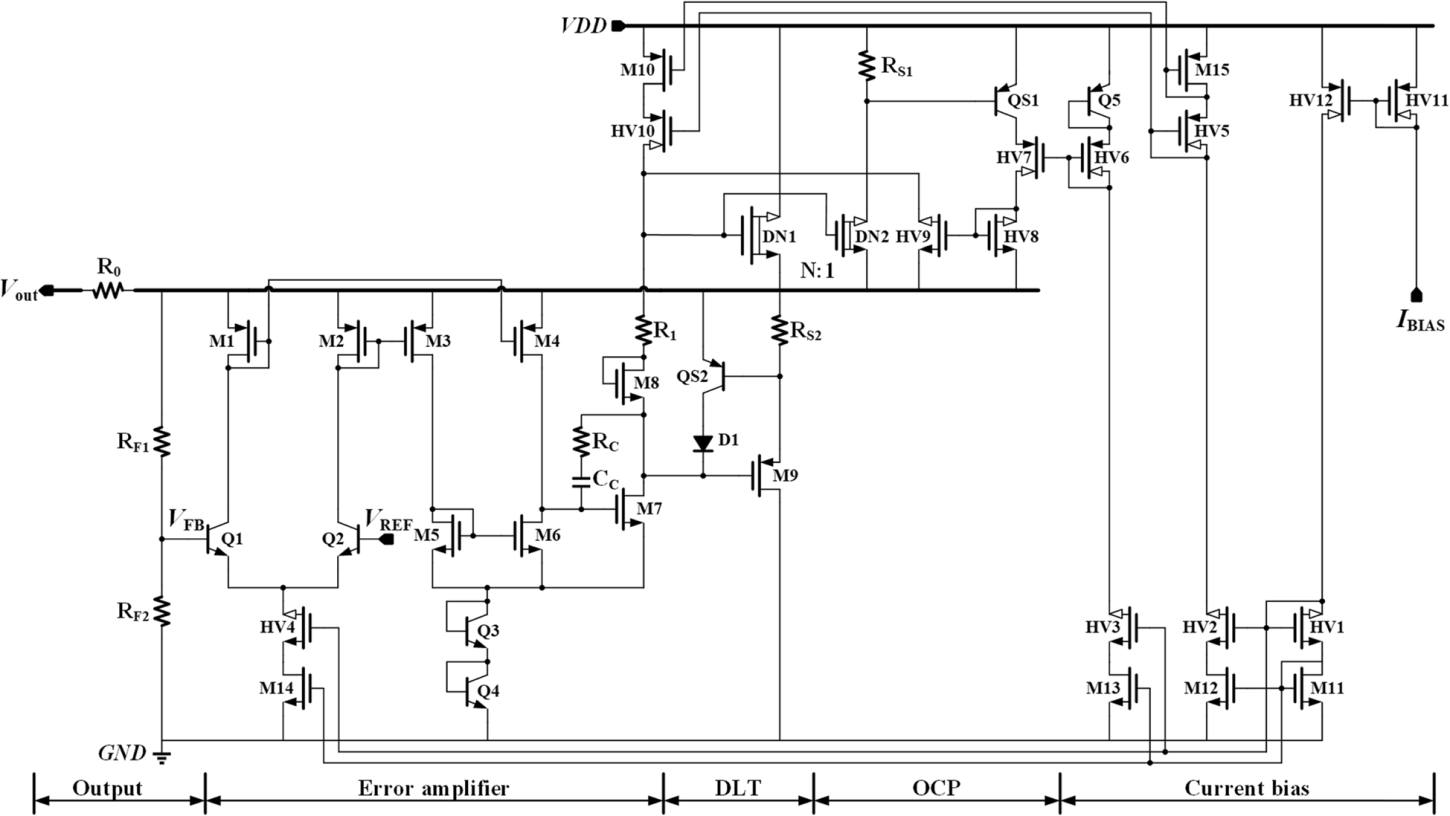
The detailed circuit of the proposed VR

The detailed circuit of the proposed VR is shown in Fig. 1.Standard low voltage MOS transistors, high voltage transistors, BJTs, N-type depletion LDMOS (laterally diffused MOS) transistors, and diodes are named M*n*, HV*n*, Q*n*, DN*n*, and D*n*, respectively in the figure, where *n* is the sequence number of the relevant device.

The proposed VR mainly includes five sub-modules: current bias, OCP, DLT, EA, and output stage. The current bias circuit provides biasing current for the whole regulator system [12, 14]. The reference voltage can be generated in many different ways [1], and the detailed circuit is not shown here. An embedded current limiting loop functions as an OCP circuit to limit the load current to a preset value. The transient enhancement circuit, which is implemented by DLT, achieves the overshoot, and undershoots suppression through adaptively changing the load current during transient procedure. The negative feedback regulation loop is formed by EA and the output power stage to adjust the output voltage. Assuming that the output voltage *V*_OUT_ and thus the feedback voltage *V*_FB_ was lower than the desired value, the gate voltage of power transistor DN1 would be pulled up with the help of the regulation loop to increase the output voltage, and vice versa. Finally, the output voltage can stabilize at

1 $$ {V}_{\mathrm{OUT}}\approx {V}_1={V}_{\mathrm{REF}}\left({R}_{\mathrm{f}1}+{R}_{\mathrm{f}2}\right)/{R}_{\mathrm{f}2} $$

In Fig. 1, it can be noticed that the output voltage of the proposed VR also powers the first gain stage of EA, which is named as SPT. With this power multiplexing technique, most of the devices in the regulation loop can be implemented by low-voltage devices. Comparing with its high-voltage counterparts, low-voltage devices have higher performance, lower cost, and smaller area, which make the proposed regulation loop achieve good regulation ability much easier. As for the second stage of EA, Q3, and Q4 are added to lift the ground supply rail, which is adopted to limit the drain-source voltage of M7, V_DS_M7_. In other words, Q3 and Q4 can prevent M7 from large voltage pressure.

For the sake of simplicity, the simplified circuit is used to illustrate the innovation ideas in the subsequent analysis.

### Proposed OCP Circuit

**Fig. 2:**
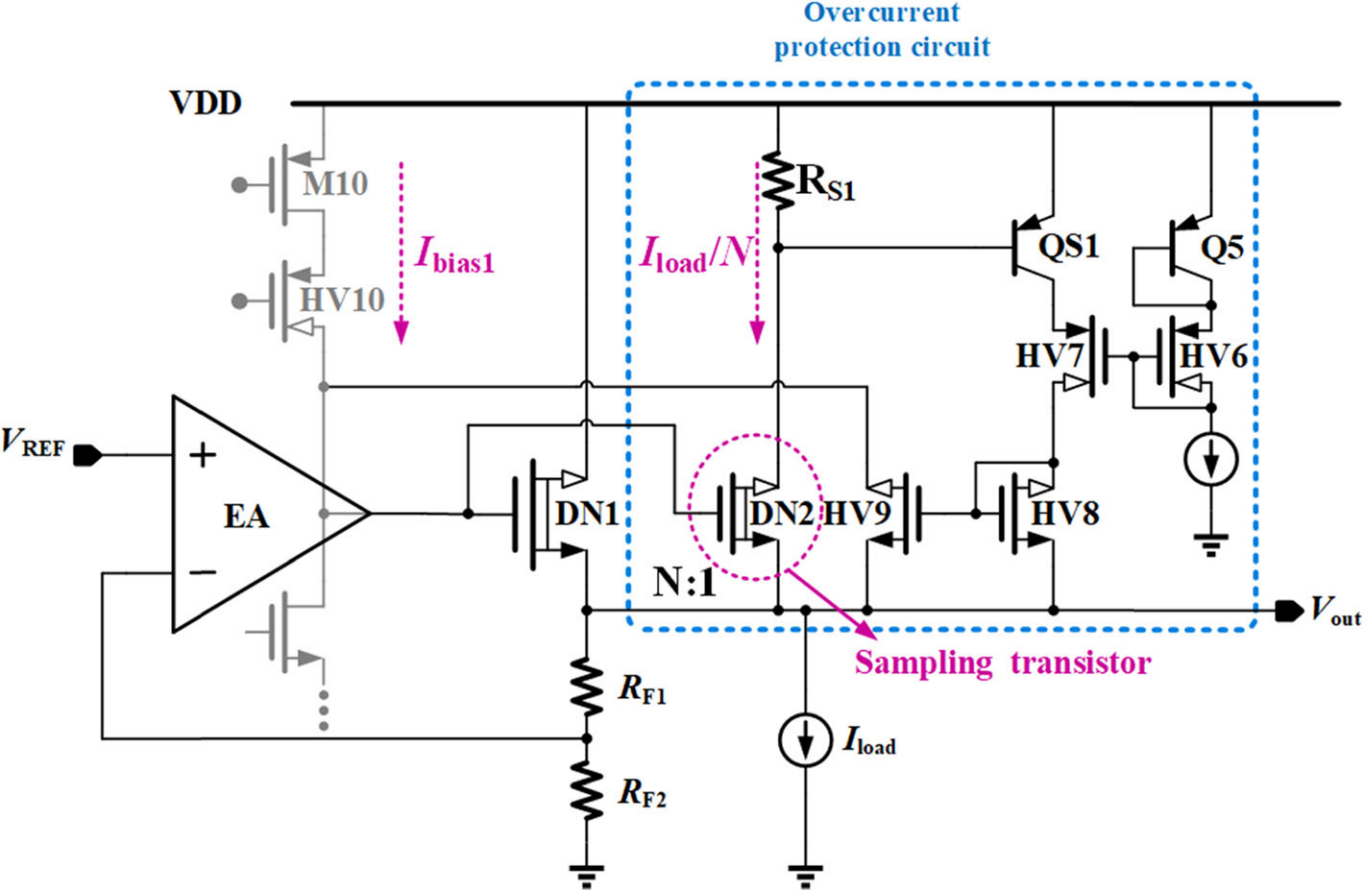
Principle of proposed OCP

Figure 2 shows the proposed OCP circuit. The proposed current limiting loop can automatically change operation mode according to different load conditions. The maximum current of proposed VR can be limited by reducing the gate voltage of the power transistor when overload occurs.

The mechanism of proposed OCP is as follows. Sampling transistor DN2 proportionally senses the current flowing through DN1 which is approximately equal to the load current *I*_Load_, which makes the voltage drop across *R*_S1_, *V*_RS1_, reflect the load current level. Once *V*_RS1_ reaches the turn-on voltage of QS1, HV8, and HV9 will form a current mirror to bypass a current from the second stage of EA. Then, the gate voltage of DN1 can be pulled down to limit the load current to a preset value, which can be expressed as

2 $$ {I}_{\mathrm{Load}}\le N\times \left({V}_{EB\left(\mathrm{QS}1\right)}/{R}_{S1}\right) $$

where *N* is the size factor ratio of DN1 to DN2. The purpose of Q5 and HV6 is to provide a proper bias voltage to HV7 and thus to protect QS1 from over-voltage condition.

There is an embedded negative feedback loop in the proposed OCP. The loop gain *T* and the dominant pole *p*_dominant_ of this current limit loop can be given by,

3 $$ T={g}_{m\_\mathrm{DN}2}{R}_{S1}{g}_{m\_\mathrm{QS}1}{R}_{\mathrm{gate}\_\mathrm{OC}} $$

4 $$ {p}_{\mathrm{dominant}}=1/\left({R}_{\mathrm{gate}\_\mathrm{OC}}{C}_{\mathrm{gate}}\right) $$

where *g*_m_DN2_ and *g*_m_QS1_ are the transconductance of DN2 and QS1, respectively. *R*_gate_OC_ ≈ (*g*_m_HV10_*r*_o_HV10_*r*_o_M10_) || *r*_o_M7_ || *r*_o_HV9_ and *C*_gate_ ≈ *C*_gs_DN1_ are the equivalent output resistance and capacitance at the gate node of power transistor DN1 when overcurrent occurs, respectively. When the proposed VR normally operates without overcurrent, HV9 is in the cutoff region, and thus, the equivalent output resistance at the gate node of DN1 named *R*_gate_ can be expressed as [(*g*_m_HV10_*r*_o_HV10_*r*_o_M10_) || *r*_o_M7_].

### Proposed DLT and Transient Enhancement Circuit

**Fig. 3:**
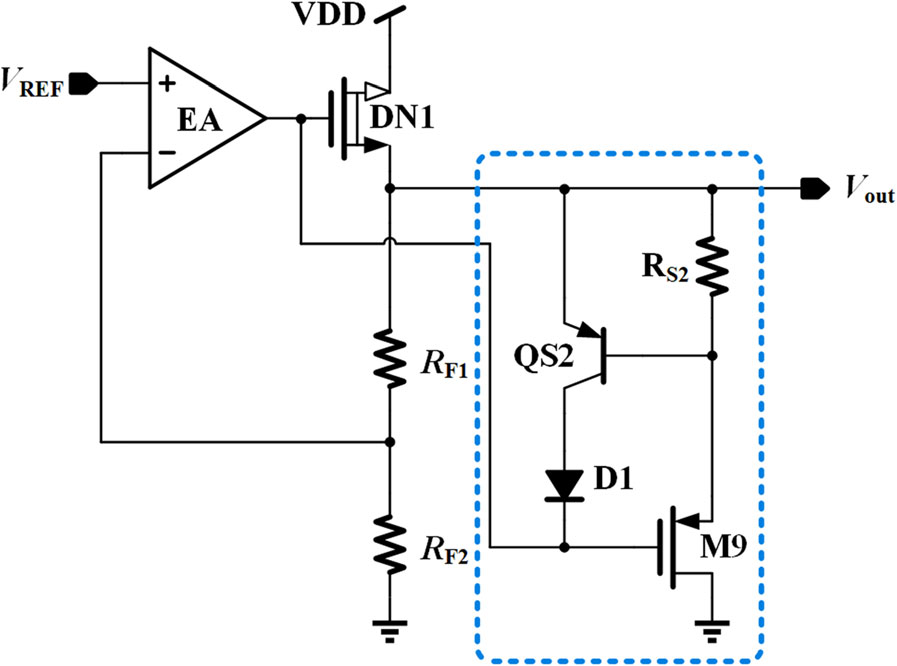
The proposed transient enhancement circuit

Figure 3 shows the transient enhancement circuit using DLT. Since the load current is proportional to Vgs_DN1 and inversely proportional to Vsg_M9, the current flowing through M9 is larger at the light load condition and is close to zero under heavy load condition. Therefore, as the load current increases, a decreased current can be introduced into the total output load. By this method, this circuit can be equalized to a dynamic load, which can be helpful to both transient enhancement and stability boosting of regulation loop.

**Fig. 4:**
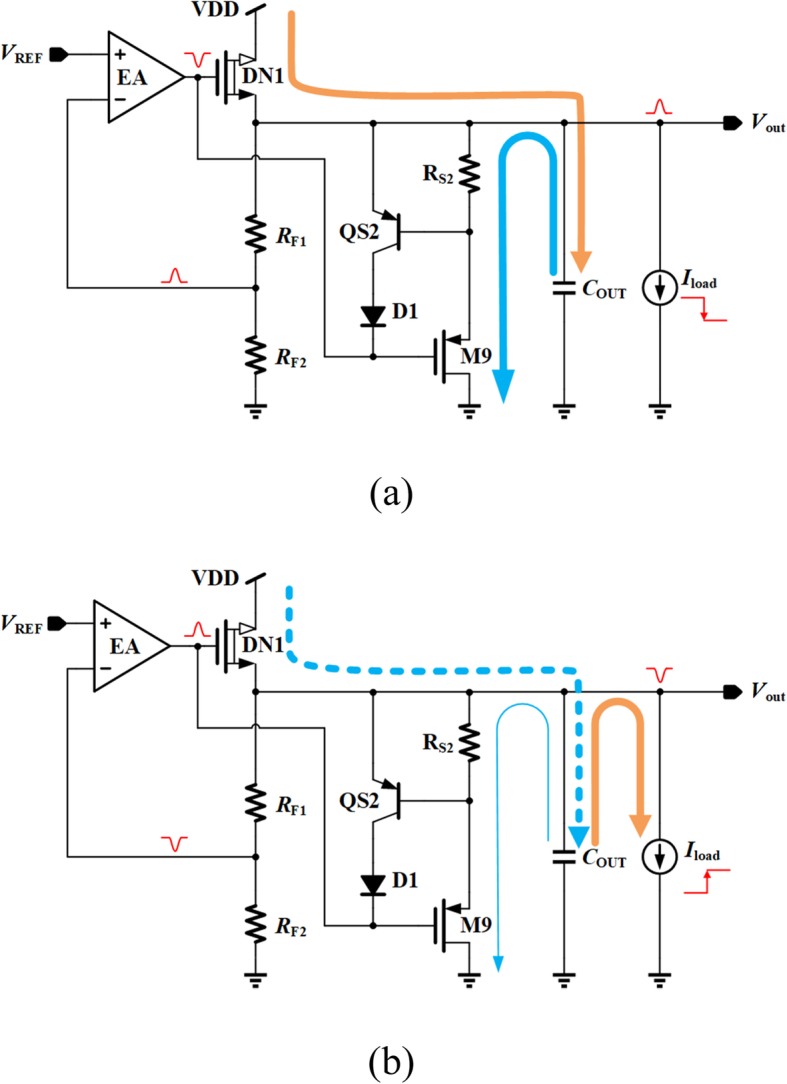
Transient response of proposed VR. **a** heavy-to-light load current change. **b** light-to-heavy load current change

The detailed transient enhancement operating principle is as follows. If the load current experiences a sudden decrease, the current flowing through power transistor DN1 will not change immediately due to the limited loop adjustment ability and slew rate. This current, shown in Fig. 4a as a yellow path, will cause an overshoot at the output voltage and thus increase the voltage drop across *R*_S2_ and M9. Then an additional current flowing through *R*_S2_ and M9, shown in Fig. 4a as a blue path, is generated at the regulated output to cancel out the unwanted yellow path current. Therefore, the output voltage spike is reduced effectively.

Figure 4b demonstrates the case of light-to-heavy load current change, where an undershoot occurs at the regulated output and then the current flowing through *R*_S2_ and M9 decreases. This can be equivalent to providing a reduced current load, so the net current flowing through the power transistor DN1 is increased, and the undershoot voltage suppression can be achieved.

To protect M9 from overcurrent, QS2 and D1 are added. When the voltage across *R*_S2_ is greater than the turn-on voltage of QS2, the extra current will flow into QS2 and D1. The maximum current in M9 is set at

5 $$ {I}_{\mathrm{M}9\_\max}\le \left({V}_{BE\_ QS2}/{R}_{S2}\right) $$

The purpose of D1 is to prevent QS2 from dropping into reversed amplifying region and flowing a reversed current in it, which is an abnormal state of the M9 current limit function.

### Stability Boosting of Proposed VR

**Fig. 5:**
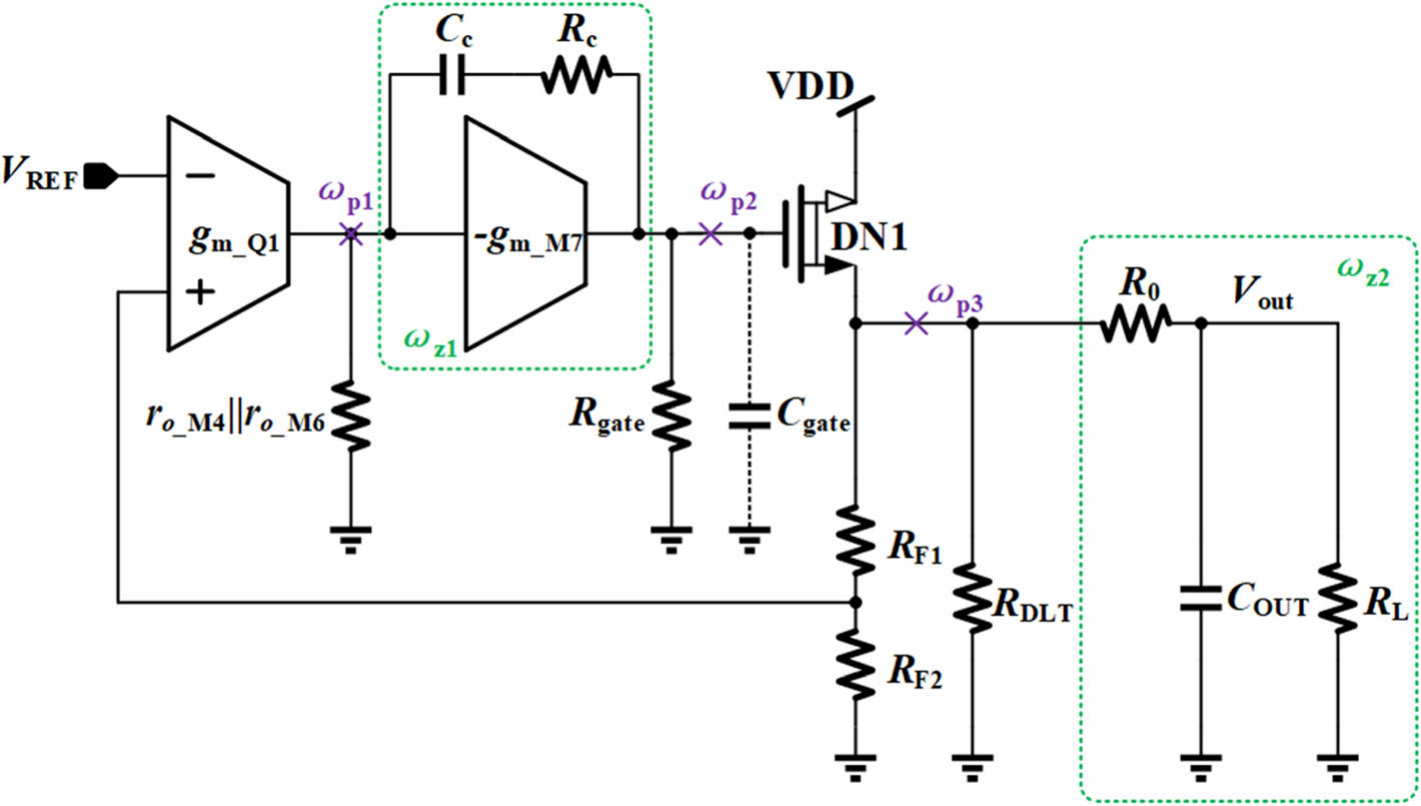
Poles and zeros distribution of proposed VR

As shown in Fig. 5, there are three poles *ω*_p1_, *ω*_p2_, and *ω*_p3_, and two zeros *ω*_z1_ and *ω*_z2_ in the control loop, and the loop gain of the proposed VR is

6 $$ {A}_{\mathrm{Vloop}}={A}_O\beta $$

where *A*_O_ is the open loop gain of the VR, and *β* is the feedback coefficient,

7 $$ {A}_O={A}_{\mathrm{CD}0}{A}_{E0}\frac{\left(1+s/{\omega}_{Z1}\right)\left(1+s/{\omega}_{Z2}\right)}{\left(1+s/{\omega}_{P1}\right)\left(1+s/{\omega}_{P3}\right)\left(1+s/{\omega}_{P2}\right)} $$

8 $$ \beta =\frac{R_{\mathrm{F}2}}{R_{\mathrm{F}1}+{R}_{\mathrm{F}2}} $$

where *A*_CD0_ ≈ 1 is the low frequency gain of the power stage that operates as a voltage follower and *A*_E0_ is the low frequency gain of the EA,

9 $$ {A}_{E0}={g}_{m\_Q1}\left({r}_{o\_M4}\Big\Vert {r}_{o\_M6}\right){g}_{m\_M7}{R}_{\mathrm{gate}} $$

Considering the Miller effect and parasitic capacitance at the gate node of DN1, the poles and zeros are written as [13]

10 $$ {\omega}_{p1}=1/\left[{g}_{m\_M7}{R}_{\mathrm{gate}}{C}_c\times \left({r}_{o\_M4}\Big\Vert {r}_{o\_M6}\right)\right] $$

11 $$ {\omega}_{p2}={g}_{m\_M7}/{C}_{\mathrm{gate}} $$

12 $$ {\omega}_{p3}=1/\left\{\left[{R}_L\Big\Vert \left({g}_{m\_\mathrm{DN}1}^{-1}\Big\Vert {R}_{\mathrm{DLT}}+{R}_0\right)\right]{C}_{\mathrm{OUT}}\right\} $$

13 $$ {\omega}_{z1}=1/\left[{C}_c\left({R}_c-1/{g}_{m\_M7}\right)\right] $$

14 $$ {\omega}_{z2}=1/\left[\left({R}_0\Big\Vert {R}_L\right){C}_{\mathrm{OUT}}\right] $$

where *R*_DLT_ is the equivalent resistance of transient enhancement circuit; *C*_OUT_ is the output capacitor of the proposed VR.

Since the compensation capacitor *C*_C_ is enlarged by (*g*_m_M7_*R*_gate_) due to the miller effect at node p1, the pole *ω*_p1_ is the dominant pole. The second pole should be *ω*_p3_, because *C*_OUT_ is usually in the range of several microfarads. Though the parasitic capacitor *C*_gate_ is relatively large, it is still smaller than both the equivalent capacitance at node p1 and the output capacitor. Besides, the resistance at node p2 is just 1/*g*_m_M7_. Hence, the pole *ω*_p2_ is located at high frequency. The zero *ω*_z1_ is to cancel mid-frequency pole *ω*_p3_. The resistor *R*_0_ generates a zero *ω*_z2_ to compensate the internal parasitic pole *ω*_p2_. The stability of the proposed VR can be improved as resistor *R*_0_ increasing. However, resistor *R*_0_ will increase the error of the output voltage due to the voltage drop caused by load current. Therefore, resistor *R*_0_ should be set in a reasonable value to make a good tradeoff between the precision of the output voltage and the loop stability.

**Fig. 6:**
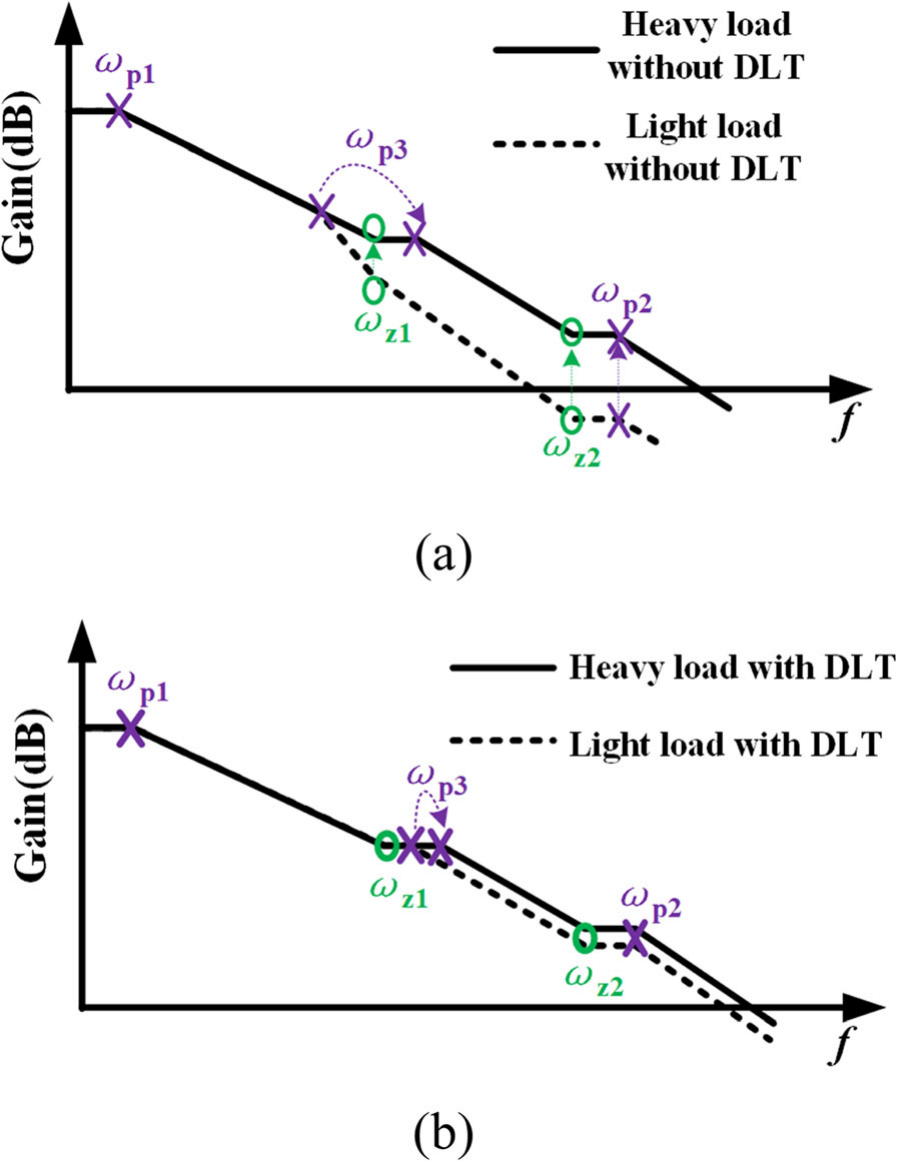
Frequency response of voltage regulator. **a** Voltage regulator without dynamic load technique. **b** The proposed voltage regulator with dynamic load technique

In a conventional voltage regulator without DLT, the pole at output node will be at different frequency because of the load current changing induced power transistor transconductance variation. As the load current increasing, the transconductance of power transistor *g*_*m*_DN1_ will increase, and thus the output pole will move towards high frequency while other zeros and poles maintaining at the same position, as shown in Fig. 6a. This may make frequency compensation of the system more difficult and slow the transient response in light load condition. More seriously, the system might be unstable.

With the help of the proposed DLT, the presented VR has better stability with different loads. As previously analyzed, the current through dynamic load circuit decreases as load current increases in steady state and vice versa. Since this current is provided by DN1, it can suppress the transconductance variations of DN1 within a wider load current range, which is helpful for the system stability and bandwidth constancy during a wider load range by using the proposed DLT. The frequency response of the proposed VR is in Fig. 6b, which can guarantee the stability with fast transient response.

The output capacitor sets the position of the zero *ω*_z2_. By placing the zero-pole reasonably, the system will have better stability with different *C*_OUT_.

### PSR Analysis of the Proposed VR

**Fig. 7:**
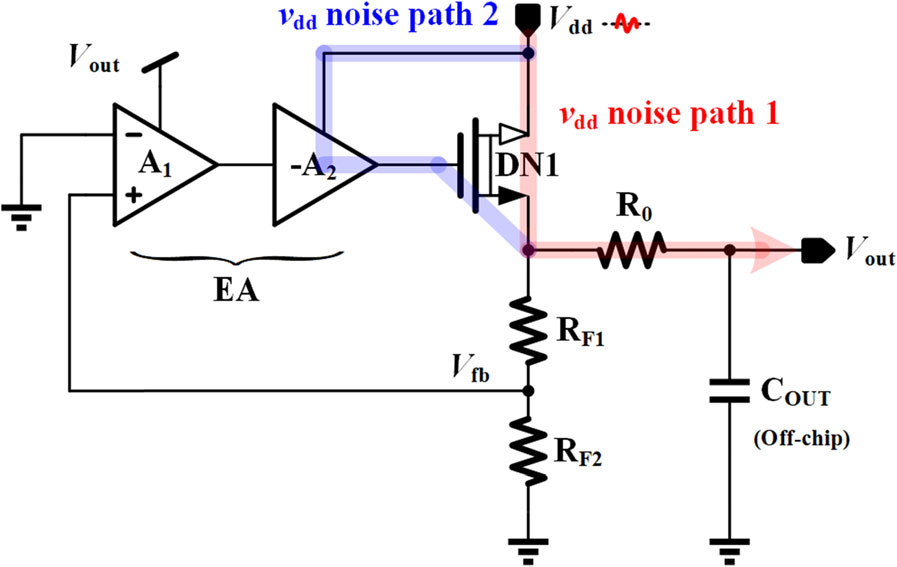
PSR analysis of the proposed VR

PSR is one of the critical parameters to measure the performance of voltage regulators, and it refers to the rejection ability against the high frequency ripples and noise arising from the supply voltage. The PSR analysis method proposed by Gupta 12 is adopted in this section, whose main idea is to simplify the whole regulator system into a voltage divider model. As shown in Fig. 7, there are two noise paths from *V*_dd_ to *V*_out_: path 1 directly transfers the noise from the drain of power transistor DN1 to *V*_out_; path 2 is from the second stage of EA to the gate of power transistor DN1. The effect of path 2 can be expressed as

15 $$ {A}_{\mathrm{path}2}\approx \frac{r_{o\_M7}}{g_{m\_\mathrm{HV}10}{r}_{o\_\mathrm{HV}10}{r}_{o\_M10}} $$

As shown in (15), *A*_path2_ is a quite small with the help of proposed SPT and cascode current mirror structure. This makes the influence of path 1 dominant in PSR analysis.

**Fig. 8:**
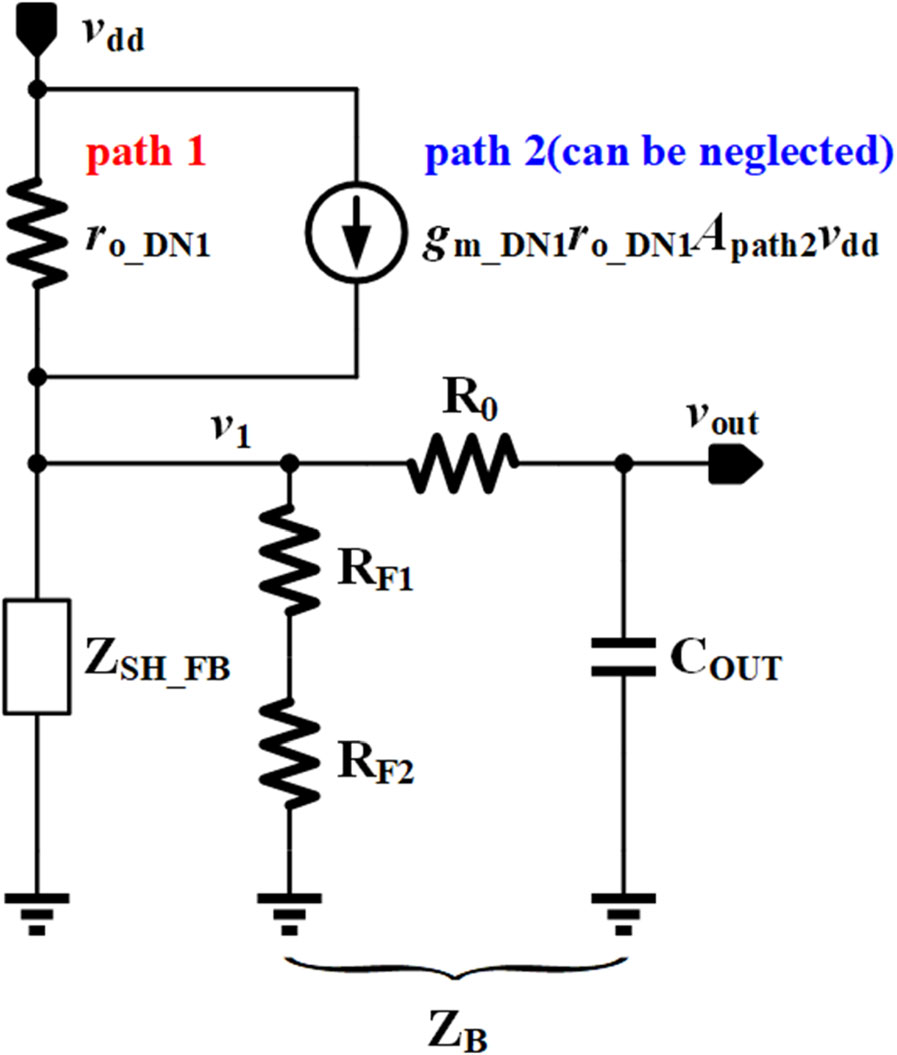
The simplified model of PSR

The simplified PSR model of the proposed VR is shown in Fig. 8, where *r*_o_DN1_ is the output resistance of power transistor DN1 accounting for noise path 1, the controlled current source originates from noise path 2, *Z*_B_ consists of *R*_F1_, *R*_F2_, *R*_0_, and *C*_OUT_ acting as a filter at high frequency, and *Z*_SH_FB_ is the equivalent impedance including the function of negative feedback loop. *Z*_SH_FB_ can be given by

16 $$ {Z}_{SH\_ FB}=\frac{1}{g_{m\_ DN1}\left(1+\beta {A}_E\right)} $$

where *g*_m_DN1_ is the transconductance of power transistor DN1. Hence, the PSR transfer function can be expressed as

17 $$ \mathrm{PSR}=\frac{V_{\mathrm{out}}}{V_{\mathrm{dd}}}=\frac{\left(1+{g}_{m\_\mathrm{DN}1}{r}_{o\_\mathrm{DN}1}{A}_{\mathrm{path}2}\right)\left({Z}_B\Big\Vert {Z}_{\mathrm{SH}\_\mathrm{FB}}\right)}{r_{o\_\mathrm{DN}1}+{Z}_B\Big\Vert {Z}_{\mathrm{SH}\_\mathrm{FB}}} $$

By deliberately setting the intrinsic gain of HV10 far greater than the power transistor DN1, *g*_m_DN1_*r*_o_DN1_*A*_path2_ < < 1 can be achieved, and thus the controlled current source can be neglected. The expression of PSR can be further simplified as

18 $$ \mathrm{PSR}\approx \frac{Z_B\Big\Vert {Z}_{\mathrm{SH}\_\mathrm{FB}}}{r_{o\_\mathrm{DN}1}+{Z}_B\Big\Vert {Z}_{\mathrm{SH}\_\mathrm{FB}}} $$

Since *Z*_B_ and *Z*_SH_FB_ will change with frequency variation, it is necessary to analyze the frequency characteristic of the PSR.

#### The Low Frequency

At low frequency, the gain of EA is very high, and *C*_OUT_ can be treated as open circuit. Thus, *Z*_B_ > > *Z*_SH_FB_ and the PSR can be written as

19 $$ {\mathrm{PSR}}_{\mathrm{LF}}\approx \frac{1}{g_{m\_\mathrm{DN}1}{r}_{o\_\mathrm{DN}1}\left(1+\beta {A}_{E0}\right)} $$

#### The Medium Frequency

The impedance of *Z*_SH_FB_ will increase because the loop gain decreases at the medium frequency. At this stage, the *Z*_SH_FB_ is still small, and the PSR is mainly affected by loop gain. With regard to (7), the PSR can be expressed as

20 $$ {\mathrm{PSR}}_{\mathrm{MF}}\approx \frac{1}{g_{m\_\mathrm{DN}1}{r}_{o\_\mathrm{DN}1}\left(1+\beta {A}_{E0}\right)}\frac{1+s/{\omega}_p}{1+s/\left[{\omega}_p\left(1+\beta {A}_{E0}\right)\right]} $$

As shown in (20), the PSR is getting worse, and the noise of output voltage is more serious while frequency increasing within unity gain frequency.

#### The High Frequency

Due to the increasing of frequency, *Z*_SH_FB_ becomes large and will finally close to 1/*g*_m_DN1_. The impedance of *C*_OUT_ becomes smaller, but it is still much larger than *R*_0_. So *R*_0_ can be omitted as before. The high frequency PSR depends on the voltage division between *r*_o_DN1_ and 1/*g*_m_DN1_ paralleled with *C*_OUT_, which can be represented by

21 $$ {\mathrm{PSR}}_{\mathrm{HF}}\approx \frac{1}{g_{m\_\mathrm{DN}1}{r}_{o\_\mathrm{DN}1}}\frac{1}{1+s{C}_{\mathrm{OUT}}/{g}_{m\_\mathrm{DN}1}} $$

At high frequency, the noise at output voltage can be suppressed greatly due to the effect of *C*_OUT_.

As previously analyzed, the good anti-noise ability in the full frequency range of proposed VR is guaranteed by three aspects. Firstly, high loop gain is adopted; Secondly, SPT makes the power supply noise have little impact on the gate of power transistor; Thirdly, the output capacitor *C*_OUT_ can improve the PSR at the high frequency with filtering property.

## Results and Discussion

**Fig. 9:**
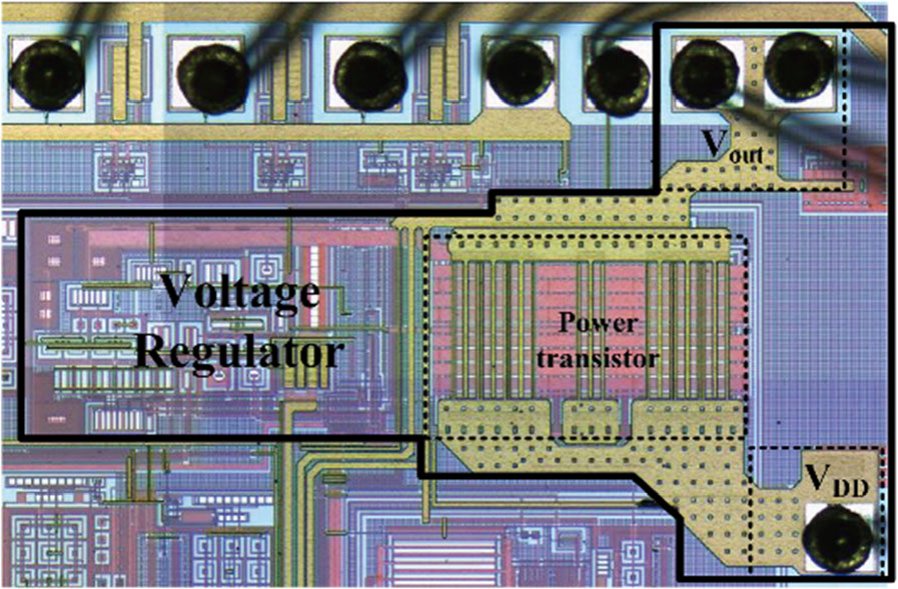
The chip photo of proposed VR

The proposed VR has been implemented in a standard 0.35-μm BCD technology. The chip photo of the fabricated regulator is shown in Fig. 9, whose active core area of the VR is 290 μm × 900 μm.

The regulated output voltage of proposed VR is 5 V with the power supply voltage ranging from 5.5 to 30 V. The output capacitor is low-cost ceramic capacitor. The capacitance of output capacitor can be set from 100 nF to 3.3 μF.

**Fig. 10:**
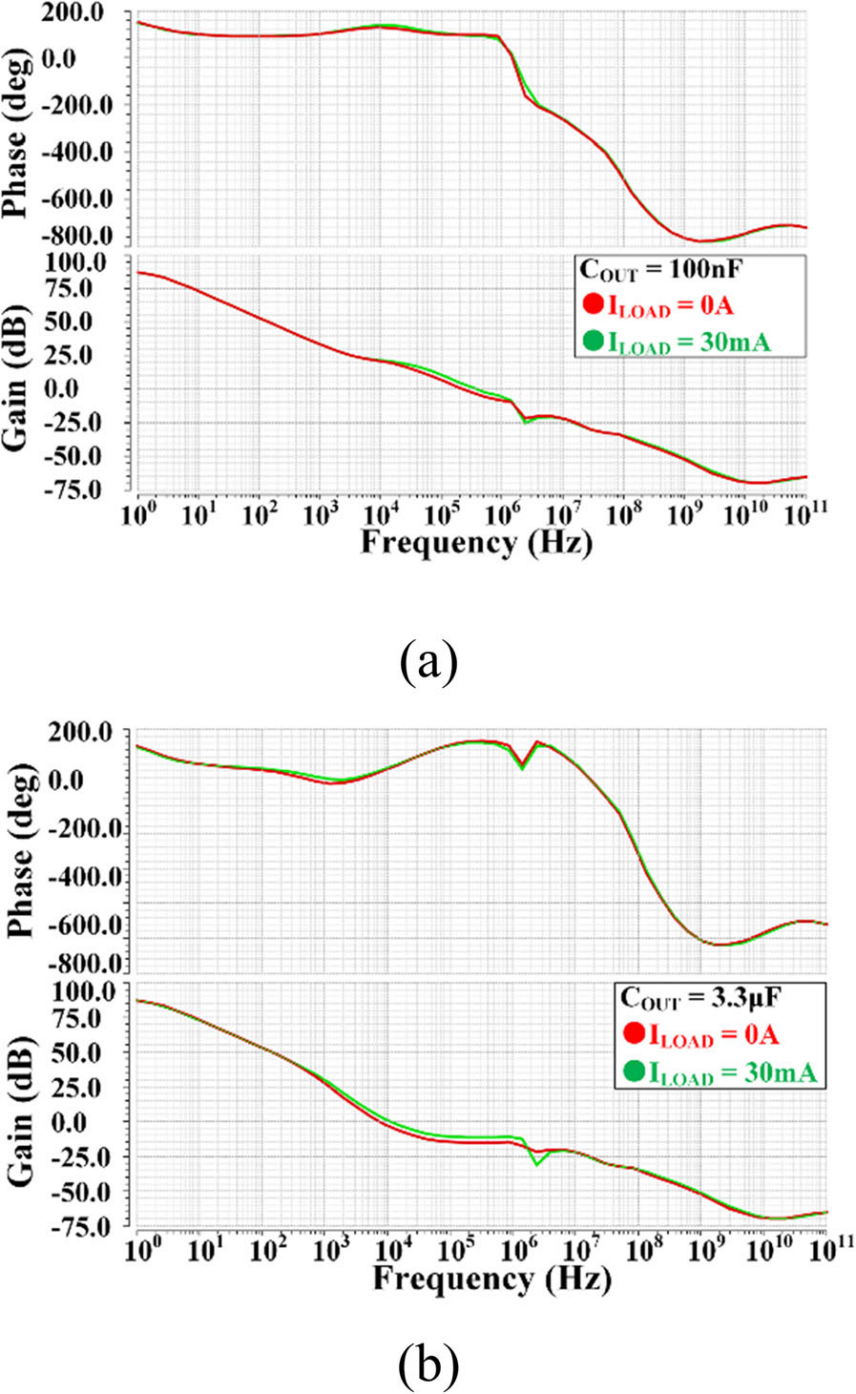
Loop frequency responses of proposed VR under different value of *I*_Load_ and *C*_OUT_ conditions. **a** C_OUT_ = 100 nF, **b** C_OUT_ = 3.3 훍F. Red and green line represents *I*_Load_ of 0 A and 30 mA, respectively

Figure 10 demonstrates the frequency response of proposed VR at different load conditions with 100 nF and 3.3 μF output capacitor. The proposed LDO can maintain stable in a wide range of output capacitor value, and the waveform of loop frequency response has very small difference between 0 and 30 mA load current, which benefits from the proposed DLT analyzed before.

**Fig. 11:**
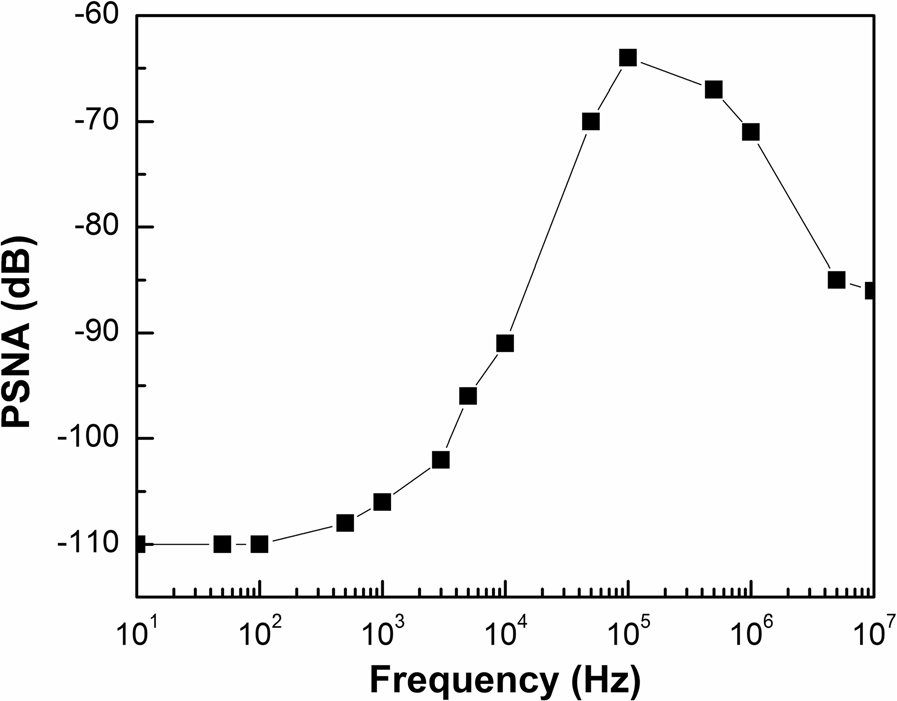
PSR of proposed VR

The PSR verification result with 0.1 μF output capacitor is shown in Fig. 11, where a − 110 dB at low frequency and better than − 64 dB up to 10 MHz is achieved. At the low frequency, the proposed VR has good PSR due to the high loop gain. The PSR become poor within unity-gain frequency because of the dominated pole *ω*_p_. The output capacitor *C*_OUT_ improves the PSR characteristic at the high frequency. Those results show that it is consistent with the previous analysis, and the proposed VR obtains better PSR in full frequency range.

**Fig. 12:**
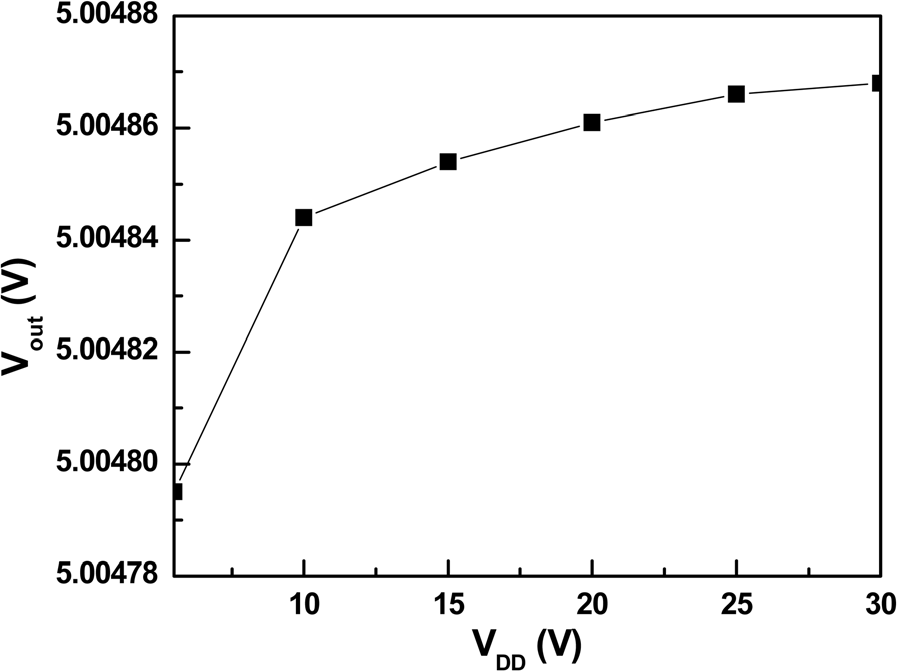
Line regulation of proposed VR

The line regulation result of proposed VR is shown in Fig. 12. In the input voltage range of 5.5 to 30 V, the output voltage only varies 73.53 μV, which results in a line regulation of only 2.98 μV/V. This confirms the effectiveness of the proposed SPT.

**Fig. 13:**
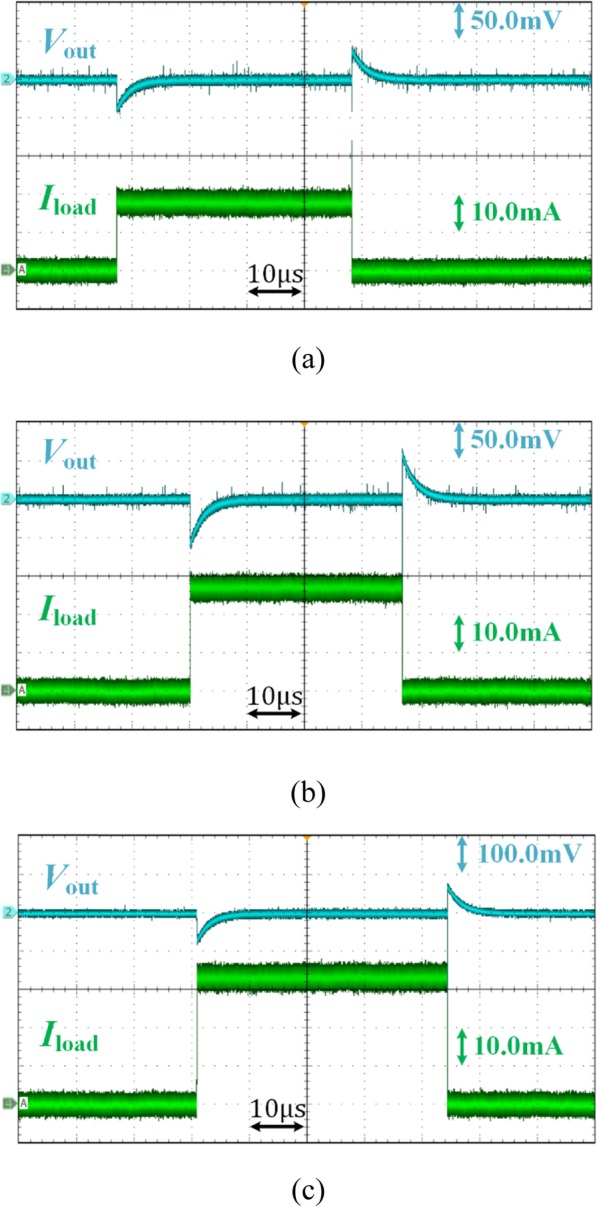
Transient response waveform of proposed VR due to different load current step. **a** 0 to 18 mA; **b** 0 to 28 mA; **c** 0 to 32 mA

Figure 13 shows the transient response of output voltage due to different load current. The voltage spike and dip of the regulated output voltage is about 43 mV, 65 mV, 83 mV when the load current changes from 0 to 18 mA, 28 mA, and 32 mA, respectively. This results in a load regulation of 0.233 mV/mA, which is mainly caused by *R*_0_ for the stability with a wide range of output capacitance.

**Fig. 14:**
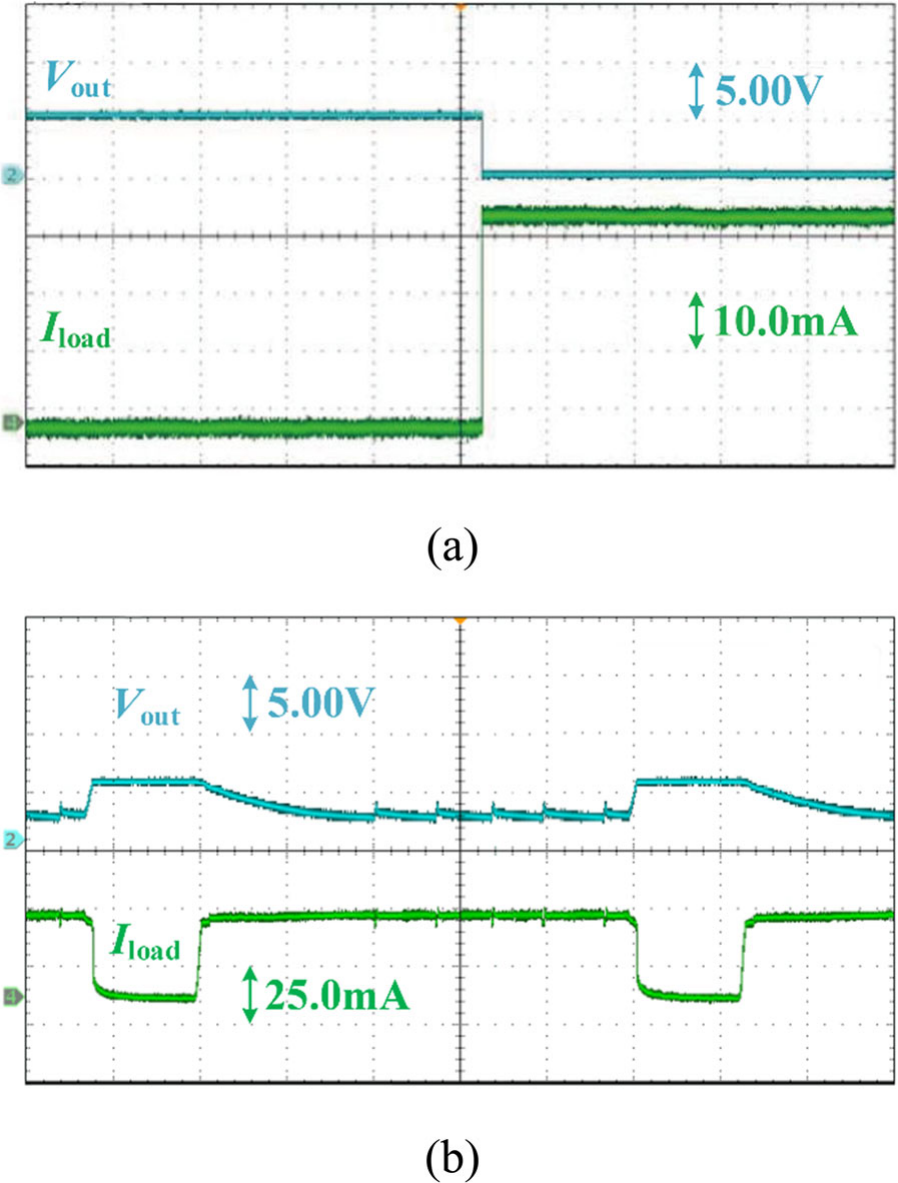
The OCP measurement of proposed VR. **a** output short circuit; **b** transient between overcurrent and normal load

The measured OCP is shown in Fig. 14. In order to verify the effectiveness of OCP, short-circuit is adopted in Fig. 14a. As shown in Fig. 14a, when the overcurrent occurs with the output voltage being pulled to ground, the output current of proposed VR is maintained at around 40 mA. Figure 14b illustrates a transient response between overcurrent and normal load, which indicates that the proposed VR has ability of self-recovery when overload exits.

Table 1 provides a performance comparison of the proposed LDO and some other previously published LDOs. In comparison, this LDO has the best line regulation and PSR, which benefits from the proposed SPT. The active area will be further reduced if fabricated in more advanced process.

**Table 1:** Performance comparison of the proposed LDO with previously published LDOs

	This work	[28]	[29]	[30]	[31]	[32]
Process (nm)	350	180	65	65	180	250
*V*_IN_ (V)	5.5–30	1.8	1–1.4	1.2	1.4 (min)	1.5 - 3.3
*V*_OUT_ (V)	5	1.2	0.8–1.2	1	1.2	1.0 - 3.3
*I*_LOAD_ (mA)	0–30	10 (max)	0–25	0.1–25	0–50	150 (max)
*C*_OUT_ (nF)	100–3300	0.2–500	0–0.025	0.12–0.54	220	1000 - 4700
Line regulation (μV/V)	2.98	N/A	700	3800	6000	N/A
Load regulation (mV/mA)	0.233	4	0.28	0.042	0.08	0.16
PSR (dB)	− 110 dB @low freq. − 86 dB@10 MHz with 0.1 μF C_OUT_	− 41 dB@1 MHz − 41 dB@10 MHz − 39 dB@20 MHz	− 26 dB@1 MHz − 11 dB@10 MHz	− 52 dB@1 MHz − 37 dB@10 MHz − 36 dB@1GHz	− 36 dB@1 MHz	>22 dB @ 0 - 20 kHz
Active Area (mm^2^)	0.261	0.079	0.0021	0.087	0.0337	0.108

Table 2 provides another performance comparison focusing on the LDOs which also have wide power supply range. With the help of the proposed DLT and SPT, this work has the best line regulation and the widest power supply range comparing with other LDOs. The additional OCP function makes this work more competitive and reliable.

**Table 2:** Performance comparison of the proposed LDO with LDOs of wide power supply range

	This work	[5]	[6]	[21]
process (μm)	0.35	0.4	0.5	1.5
*V*_IN_ (V)	5.5–30	3.9–20	4.5–28	5.7–30
*V*_OUT_ (V)	5	2.5	1.8	6
*I*_LOAD_ (mA)	0–30	0–1500	20 (max)	100 (max)
*C*_OUT_ (μF)	0.1–3.3	2.2	2.1	N/A
Line regulation (μV/V)	2.98	N/A	8.3	10870
Load regulation (mV/mA)	0.233	0.0013	0.0026	0.094

## Conclusion

A high stability SPT VR with DLT and OCP is implemented in a standard 0.35-μm BCD process. With the help of SPT, most of the regulation loop is supplied by a regulated output voltage, which is beneficial for stability and PSR improvement. The proposed DLT is helpful to transient response and stability. Besides, the embedded OCP circuit can prevent the presented VR from damage by overload or short circuit. The linear regulation of the proposed VR is 2.98 μV/V with VDD from 5.5 to 30 V while the regulated output voltage is 5 V, and the load regulation is 0.233 mV/mA with load current from 0 A to 30 mA. The overshoot and undershoot voltage during load current changing is also small by using the presented transient enhancement circuit. The PSR at low frequency is − 110 dB, and is better than − 64 dB up to 10 MHz. High loop stability can be achieved in a wide range of output capacitor and load current, and thus the proposed VR is suitable for applications that require high performance and reliability under variations of output capacitor and load current.

## Data Availability

All data generated or analysed during this study are included in this published article.

## Abbreviations

VR: Voltage regulator
OCP: Overcurrent protection
SPT: Self-power technique
PSR: Power supply rejection
DLT: Dynamic load technique
EA: Error amplifier
